# STINGing the immune system: lessons learned through a model of G34-mutant pediatric high-grade glioma

**DOI:** 10.1172/JCI164420

**Published:** 2022-11-15

**Authors:** Connor P. Hall, James C. Cronk, Jeffrey A. Rubens

**Affiliations:** 1 Division of Pediatric Oncology, Sidney Kimmel Comprehensive Cancer Center, Johns Hopkins University, School of Medicine, Baltimore, Maryland, USA.; 2 Pediatric Oncology Branch, Center for Cancer Research, National Cancer Institute, NIH, Bethesda, Maryland, USA.

## Abstract

Pediatric high-grade gliomas (pHGGs) are aggressive diseases with poor outcomes. The diverse molecular heterogeneity in these rare tumors and inadequate tumor models have limited the development of effective therapies. In this issue of the *JCI*, Haase et al. produced a genetically engineered mouse model of H3.3-G34R–mutant pHGG to help identify vulnerabilities in DNA repair pathways. The authors designed a therapy that combined radiation with DNA damage response inhibitors to induce an adaptive immune response and extend survival. These findings suggest that combinations of small-molecule therapies with immunotherapies could drive a more durable response and improve mortality for patients with pHGG.

## Evolving landscape of pediatric high-grade gliomas

CNS tumors are the second most common neoplasms in children but the number one cause of cancer-related deaths (1). While there have been remarkable advancements in therapies for childhood cancers over the past 50 years, treatments for CNS tumors have lagged, and survival rates have only seen moderate gains (2). Recent advancements in our understanding of pediatric brain tumors have identified considerable molecular heterogeneity within broad categories of tumors and distinct drivers and characteristics of pediatric brain tumors compared with histologically similar tumors affecting adults (3). These advancements must be considered when designing therapies that specifically target more homogenous subgroups of tumors to improve survival for patients with these deadly diseases.

Pediatric high-grade gliomas (pHGGs) illustrate these challenges in treating childhood CNS tumors. pHGGs make up approximately 10% of pediatric CNS tumors but are responsible for 40% of CNS tumor–related deaths (2). Recent studies have demonstrated that pHGGs are distinct from their adult counterparts and consist of a heterogeneous group of tumors with a variety of biological drivers and responses to therapy (4). The most recent WHO classification of CNS tumors divides pHGGs into 4 subgroups: H3 K27-altered, H3-G34–mutant, H3 wild-type and IDH wild-type, and infant-type hemispheric gliomas (5). However, standard therapies are the same for all pHGGs and are largely derived from treatments for adult HGG. As a result, the 5-year overall survival rate remains less than 20% (6).

## Molecular mechanisms of G34-mutant pHGG

In this issue of the *JCI*, Haase et al. make important advancements toward improving therapies for pHGG by identifying unique pathway vulnerabilities in the H3-G34–mutant subgroup of pHGGs. The authors then designed and characterized a therapy for targeting these tumors (7). The H3-G34–mutant subgroup of pHGG accounts for approximately 16% of all hemispheric pHGGs, which usually affect adolescents and young adults, and can coexist with inactivating mutations in P53 and ATRX (4, 8, 9). The prognosis for this subgroup is moderate compared with other pHGG tumors, favorable compared with H3 K27–altered tumors, but unfavorable compared with the H3 wild-type and IDH wild-type subgroup (4). Mutations in histone 3.3 at glycine 34 result in an arginine (G34R) or, less commonly, a valine (G34V) substitution, but the biological effects of the mutation are not yet well understood (10).

**Figure 1:**
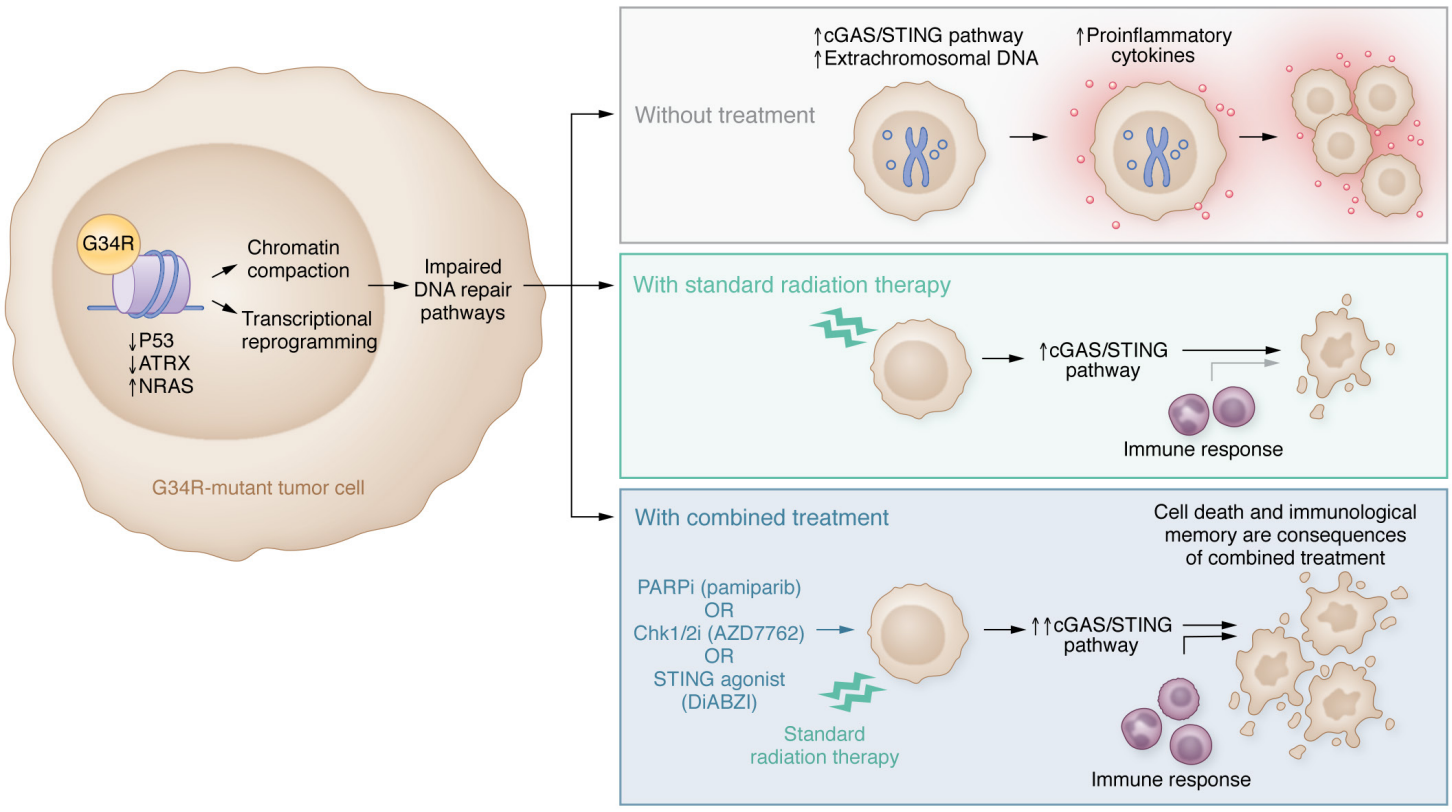
Targeting vulnerabilities in DNA damage response pathways in G34R-mutant pHGG induces cell death and immunological memory. DNA repair pathways are downregulated in G34R-mutant pHGGs, leading to increased DNA damage and activation of the cGAS/STING immune response. Radiation combined with DNA damage response inhibitors or a STING agonist leads to cell death and immunological memory, and extends survival in models of G34R-mutant pHGG. Chk1/2i, Chk1/2 inhibitor; PARPi, PARP inhibitor.

To better characterize these tumors, Haase and colleagues developed a genetically engineered mouse model (GEMM) to mimic the biological characteristics of H3.3-G34R–mutant tumors and clarify their driving molecular mechanisms. They compared gene expression in this model with an H3.3 wild-type GEMM and also compared an H3.3-G34R–mutant human cell line with an H3.3 wild-type cell line. DNA repair pathways were downregulated in G34R-mutant tumors, which increased DNA damage, the accumulation of extrachromosomal DNA, and activated the cyclic GMP-AMP synthase/stimulator of IFN gene signaling (cGAS/STING) pathway to induce the release of proinflammatory cytokines (Figure 1). The authors then targeted these abnormalities in the DNA repair pathway by combining standard radiation therapy with DNA damage response inhibitors. The PARP inhibitor pamiparib combined especially effectively with radiation, resulting in more than 50% long-term survivors beyond 90 days compared with a median survival of 32, 39, and 44 days in the nontreated, pamiparib alone, and radiation alone treatment arms, respectively (7).

Notably, there has already been a Children’s Oncology Group–led (COG-led) clinical trial (ACNS1721) testing the PARP inhibitor veliparib in combination with radiotherapy for pHGG, which was closed early due to poor outcomes compared with historical controls (11). However, Haase et al. noted that veliparib showed weaker potency compared with pamiparib, and veliparib was ineffective when combined with radiation to treat their models of pHGG (7). The COG trial also highlights the importance of developing treatments for the more homogenous subgroups of tumors, as we would not expect the same DNA repair defects or similar responses to PARP inhibitors in other subgroups of pHGG. While the number of patients enrolled in ACNS1721 may be inadequate to make broad conclusions, it would be interesting to assess whether patients with G34-mutant HGG responded any better to combination therapy in this trial (11).

## Cancer models and the tumor microenvironment

Inadequate tumor modeling has contributed to the limited efficacy of translating preclinical studies into successful clinical trials (12). One of the major gaps in many preclinical studies is the use of immunocompromised host organisms. It has been recognized in recent years that the immune system is critical to the successful treatment of cancers. The development of immunotherapy has enormously improved cancer outcomes. In addition, immune responses contribute to the success of more traditional therapies like chemotherapy and radiation as well as of treatments using many small-molecule inhibitors (13). While these therapies are often designed for their direct cytocidal effects, they also have an important impact on the tumor microenvironment including influencing resident and recruited immune cells (13). In contrast, some small-molecule inhibitors may tamp down the immune response, thereby limiting their efficacy. Immunocompromised preclinical models of cancer likely miss these important treatment effects and may not accurately predict the effects of therapy on human cancers.

Haase et al. address this issue with the development of their GEMM model of pHGG. GEMM tumor models were specifically designed to recapitulate the genetic drivers of human cancers that arise de novo within the mouse. However, these models are not without limitations. The GEMM model of pHGG was created on the basis of brain neural precursor cells incorporating the H3.3-G34R mutation, knockdown of *p53* and *Atrx* using shRNA, and expression of constitutively active NRAS to represent RTK/RAS/PI3K activation (7). However, some human H3.3-G34R/V tumors may not have inactivating mutations in P53 and/or ATRX, and the mechanism of RTK/RAS/PI3K activation is more variable (4). From a biological standpoint, it is difficult to know if this model accurately represents H3.3-G34R tumors, a minor subset of this group, or is entirely distinct from human tumors. An alternative approach to modeling these tumors could include humanized mice, that is, immunocompromised mice that have been engrafted with a partial human immune system. However, humanized mice are complex to produce, expensive, and carry other limitations, such as the development of graft-versus-host disease (14). Traditional syngeneic tumor models also have the benefit of a competent immune system but may not faithfully model a true primary tumor microenvironment or immune response, given the necessity of orthotopic injection of preexisting tumor cells (15).

## Immune responses to precision therapies

The immunocompetent model used by Haase et al. allowed the authors to identify the activation of the cGAS/STING pathway in G34-mutant pHGG after radiation therapy and the subsequent increase in secreted IFN-β. Strikingly, the authors demonstrated that activation of the cGAS/STING pathway led to immune memory by showing that rechallenging mice with tumors implanted into the contralateral cerebral hemisphere did not lead to new tumor growth. These results show that radiation combined with pamiparib not only caused cytocidal effects on cancer cells but also contributed to an adaptive immune response that has the potential for longer-term benefits (7).

Precision therapies and immunotherapies are often considered to be two distinct pathways to improve cancer treatments. However, an improved understanding of the immune consequences of precision therapies like PARP inhibitors suggest that there may be untapped synergies in combining these categories of therapies to improve outcomes. Checkpoint inhibitors effectively treat many cancers by combating cancer-driven immunosuppression and T cell exhaustion of the immune system, however the efficacy of checkpoint inhibitors has been limited in the treatment of pediatric cancers (16, 17). The results reported in the study by Haase and colleagues hint that immune stimulation from radiation and PARP inhibitors in G34-mutant pHGG could lead to potential synergies in combination with checkpoint inhibitors to further amplify the immune response against these cancers and improve survival (7). In fact, preclinical studies and clinical trials are starting to emerge using a similar treatment approach with some early success (18–20). Further studies designed to understand the immune consequences of targeted treatments may help researchers develop additional combination therapies harnessing the immune system to overcome therapy resistance and drive more durable adaptive immune responses to improve survival.

## Conclusion

The Haase et al. study highlights the value of the GEMM model in identifying targetable pathways and testing candidate therapies in an immunocompetent host and supports the growing body of evidence for tailor-made therapies based on the molecular stratification of tumors in pediatric neuro-oncology (7). It remains to be seen if the therapeutic approaches of radiation therapy combined with PARP inhibition, cell-cycle checkpoint inhibition, and cGAS/STING agonist therapy will translate to the clinic, however, this approach holds promise for a group of patients in dire need of better treatments.

## Version 1 11/15/2022

Electronic publication
